# Annotating TSSs in Multiple Cell Types Based on DNA Sequence and RNA-seq Data via DeeReCT-TSS

**DOI:** 10.1016/j.gpb.2022.11.010

**Published:** 2022-12-15

**Authors:** Juexiao Zhou, Bin Zhang, Haoyang Li, Longxi Zhou, Zhongxiao Li, Yongkang Long, Wenkai Han, Mengran Wang, Huanhuan Cui, Jingjing Li, Wei Chen, Xin Gao

**Affiliations:** 1 Computer Science Program, Computer, Electrical and Mathematical Sciences and Engineering Division, King Abdullah University of Science and Technology, Thuwal 23955-6900, Saudi Arabia; 2 Computational Bioscience Research Center, King Abdullah University of Science and Technology, Thuwal 23955-6900, Saudi Arabia; 3 Department of Biology, School of Life Sciences, Southern University of Science and Technology, Shenzhen 518055, China; 4 Shenzhen Key Laboratory of Gene Regulation and Systems Biology, School of Life Sciences, Southern University of Science and Technology, Shenzhen 518055, China; 5 Academy for Advanced Interdisciplinary Studies, Southern University of Science and Technology, Shenzhen 518055, China

**Keywords:** Transcription start site, Machine learning, Deep learning, Meta-learning, RNA sequencing

## Abstract

The accurate annotation of **transcription start sites** (TSSs) and their usage are critical for the mechanistic understanding of gene regulation in different biological contexts. To fulfill this, specific high-throughput experimental technologies have been developed to capture TSSs in a genome-wide manner, and various computational tools have also been developed for *in silico* prediction of TSSs solely based on genomic sequences. Most of these computational tools cast the problem as a binary classification task on a balanced dataset, thus resulting in drastic false positive predictions when applied on the genome scale. Here, we present DeeReCT-TSS, a **deep learning**-based method that is capable of identifying TSSs across the whole genome based on both DNA sequence and conventional **RNA sequencing** data. We show that by effectively incorporating these two sources of information, DeeReCT-TSS significantly outperforms other solely sequence-based methods on the precise annotation of TSSs used in different cell types. Furthermore, we develop a **meta-learning**-based extension for simultaneous TSS annotations on 10 cell types, which enables the identification of cell type-specific TSSs. Finally, we demonstrate the high precision of DeeReCT-TSS on two independent datasets by correlating our predicted TSSs with experimentally defined TSS chromatin states. The source code for DeeReCT-TSS is available at https://github.com/JoshuaChou2018/DeeReCT-TSS_release and https://ngdc.cncb.ac.cn/biocode/tools/BT007316.

## Introduction

The transcription start site (TSS) is the first nucleotide where a gene is transcribed [1]. Promoter regions around TSSs usually contain multiple *cis*-elements that can be recognized by transcription factors (TFs) recruited by polymerase [2]. Therefore, precisely locating TSSs and their associated promoter regions is essential for understanding the *cis*–*trans* networks of transcriptional regulation [3].

To fulfill this, specific high-throughput experimental technologies have been developed to capture TSSs in a genome-wide manner. As mature transcripts produced by RNA polymerase II (RNAPII) have a specific cap structure at their 5′ ends, a so-called cap analysis of gene expression (CAGE) method has been developed to capture the TSSs [4]. By integrating the CAGE method with massive parallel sequencing (CAGE-seq), the method can identify TSS positions and quantify the usage in a genome-wide manner. In the last two decades, Functional Annotation of The Mammalian Genome (FANTOM), a worldwide collaborative project aiming at identifying all functional elements in mammalian genomes, has performed CAGE-seq in dozens of cell lines and primary tissues [5]. At the chromatin level, promoter regions are enriched with specific histone post-translational modifications (PTMs), including H3K4me3, H3K9ac, and H3K27ac. In addition, DNA regions around TSSs are usually unbound by nucleosomes, therefore manifested as nucleosome-depleted regions (NDRs). Together, such NDRs flanked by regions enriched in H3K4me3, H3K9ac, and H3K27ac modifications could be used to represent a promoter-specific chromatin state [6]. In the ENCyclopedia Of DNA Element (ENCODE) project, chromatin immunoprecipitation followed by high-throughput sequencing (ChIP-seq) has been applied to characterize a series of histone modification patterns in the human genome. Using a hidden Markov model, these data enable the prediction of different chromatin states, including the promoter-specific ones [7].

In addition to the experimental approach, many computational methods have been developed to predict TSSs by learning features in the flanking regions of the annotated TSSs. Two major strategies have been employed in promoter/TSS prediction.

One focuses on manual extraction of TSSs and promoters related *cis*-elements, and improves the performance by extending the predefined feature sets. For example, TSSG and TSSW [8] consider the TATA-box score, *cis*-element preferences, and potential *trans*-factor binding sites [9]. PromH extends the feature set of TSSW by taking into account conserved features of major promoter functional components, including transcription start points, TATA-boxes, and regulatory motifs, in pairs of orthologous genes, to further improve the promoter/TSS prediction accuracy [10].

The second strategy applies machine learning models to automatically extract features to predict promoters/TSSs. Promoter2.0 is the seminal work that uses neural networks in the promoter identification [11], and DragonGSF further uses the information of GC contents and CpG islands together with neural networks to identify promoter regions [12]. Recently, deep learning based on convolutional neural network (CNN) has been applied to the task, including CNNProm [13], TSSPlant [14], PromID [15], TransPrise [16], and iPSW(PseDNC-DL) [17], which significantly outperform previous traditional manual feature set-based tools on their target problems probably due to their powerful ability to automatically extract sequence features, especially for the large number of new features that are not included in those manual feature sets. Importantly, the majority of machine learning-based methods for TSS prediction can only solve the binary classification task on balanced datasets and cannot be applied for genome scanning due to the extreme data imbalance. So far, only PromID is able to scan the TSSs in the small regions around known TSSs (−5000 bp to +5000 bp) and claims the ability to scan promoters/TSSs on a genome scale.

More importantly, even though the DNA sequence of a promoter is identical across different types of cells, it might be activated in only certain cell types, which shapes a cell type-specific TSS landscape. However, all of the previous computational methods only consider the DNA sequence information and therefore are apparently unable to determine whether a TSS is active or inactive in a given cell type. Although experimental CAGE-seq or chromatin state-based methods like ChIP-seq are able to identify active TSSs by profiling multiple histone modification markers, they are rather laborious and costly to perform on a grand scale. As a result, other than FANTOM, ENCODE, and a couple of epigenome roadmap projects, the CAGE-seq or histone modification data with ChIP-seq have only been sparsely collected. In contrast, conventional RNA sequencing (RNA-seq) has been routinely used to characterize transcriptional profiles across tremendous amounts of primary tissues and cell lines. Thus, for computational methods to predict active TSSs in a biological sample, it would be convenient to integrate the conventional RNA-seq data with the DNA sequence information to further improve the performance in predicting TSSs precisely. On one hand, RNA-seq data would provide a distinct coverage pattern at a TSS flanking region. Theoretically, across a TSS, there should manifest a sharp increase of RNA-seq coverage. On the other hand, since only expressed regions need to be scanned, by integrating RNA-seq data, the total number of sites for genome-wide TSS prediction could be dramatically reduced.

Here, we introduce DeeReCT-TSS, a novel deep learning-based method for the accurate prediction of TSSs by incorporating both DNA sequence and RNA-seq coverage information as a new member in the Deep Regulatory Code and Tools (DeeReCT) family [18], [19]. For any sample with conventional RNA-seq data, our method could predict active TSSs in a genome-wide manner. Furthermore, by extending our method through meta-learning, we use DeeReCT-TSS for simultaneous TSS annotations on 10 cell types, and find that DeeReCT-TSS is able to identify cell type-specific TSSs. After extracting sequence features from the model, putative TFs regulating these cell type-specific TSSs could be identified. Finally, we validate the generalization power of the model by showing that the predictions from two independent ENCODE datasets are highly consistent with the TSSs identified based on chromatin states.

## Method

### Deep neural networks for binary classification

We designed a deep neural network to extract information from both the DNA sequence and the RNA-seq coverage, and the details of data preparation are shown in File S1A. Both inputs went through two independent CNNs that had a convolution layer with 64 filters of width 10 as the first layer to extract motif features and the trend of coverage changes. Following the convolution layer, a rectified linear unit (ReLU) was applied as the activation function followed by the max pooling layer. Two feature vectors were concatenated together and fed into the fully connected (FC) layer. The last softmax layer gave the prediction between 0 and 1. Weight decay and dropout were applied to improve the generalization capability of our method. We used TensorFlow 1.14.0 as the framework of our model, trained the model, and applied the model for prediction with one Tesla V100 GPU on average 2 h for 10,000 epochs and around 30 min for one human sample.

Following the work in PromID, we chose 1001 as the length of both the DNA sequence and the corresponding RNA-seq coverage inputs, in order to maintain a balance between retaining enough information and reducing computing resource requirements as longer input means more potential features and greater memory and time requirements. The DNA sequence was one-hot coded with the dimension of 1 × 1001 × 4, in which A was encoded as (1 0 0 0), T was encoded as (0 1 0 0), C was encoded as (0 0 1 0), and G was encoded as (0 0 0 1). The coverage information was strand-sensitively counted based on the RNA-seq data with SAMtools [20]. Then, RNA-seq coverage information was in the dimension of 1 × 1001 × 1 and directly used without preprocessing as one of inputs to keep the original information representing the expression levels. Both the DNA sequence and the RNA-seq coverage were fed into the network with the same architecture, resulting in the predicted value for each site in each TSS peak. The preparation of positive and negative datasets from different cell types is explained in detail in File S1A.

### Circular training for genome scanning

Binary classification is built on balanced datasets, whereas the data in genome scanning are highly unbalanced. Thus, we introduced the iterative negative data enhancement as a negative data argumentation method to our model to reduce the false positive rate (FPR) as shown in Algorithm 1 (File S1B). In brief, for each repetition, we trained the model and selected the best one by evaluating with multiple metrics, including recall, false discovery rate (FDR), and F1-score. Then we randomly selected 100 true TSS positions (+1) from the training dataset and applied the best model on the scanning task in the corresponding scanning window (−5000 bp to +5000 bp). For each scanning window, we divided it into 2 partitions. One was the positive region around the true TSS site (−500 bp to +501 bp, with TSS locate at +1), and all others outside the positive region were considered as negative regions. Any site predicted as a TSS in the positive region will be considered as true positive (TP), otherwise false positive (FP). Then half of the negative data in the training dataset was replaced by the randomly selected cases from FPs generated from the scanning task, and the whole process was repeated again for the next repetition. In this way, the results of the initial round were irreverent to the circular training, so we could compare the results of the initial round with subsequent rounds as an ablation study for applying the circular training. Since the positive and negative samples in the genome are extremely unbalanced, the selection of the same number of negative samples as the ground truth (negative TSSs) in the initial round could be not representative enough, which might generate a large number of FPs during genome scanning. Therefore, by using the circular training, the trained model could be able to perform a more accurate TSS classification and reduce the FPR in genome scanning because continuously adding the misclassified data to the training dataset makes the model remember more complicated features from the negative samples.

### Meta-learning across multiple cell lines

To obtain a more generalized model across different cell types, we incorporated the state-of-the-art meta-learning algorithm and trained the meta-model from 10 cell lines. We integrated the Reptile [21] algorithm to achieve the meta-learning across multiple cell types as shown in Algorithm 2 (File S1C). Given the meta-model from 10 cell types, we further fine-tuned with 20% data of the corresponding cell type respectively, and obtained the cell type-specific model.

### Hyperparameter and model selection

To ensure that all models had a fair chance of learning a useful representation, we trained multiple instances of each model on the binary classification task using manually generated hyperparameter settings, including learning rate, number of epochs, number of hidden layers, number of filters, batch size, momentum, initial weight, weight decay, and keep probability. Then, we selected model instances based on their training performance and fixed the hyperparameters for all following models as below for easier reproduction of the training process: number of epochs = 10,000, batch size = 4000, learning rate = 1E−4, momentum = 0.98, weight decay = 4E−4, and keep probability = 0.5.

### Evaluation metrics

To evaluate our method and to objectively compare predictions by our model and other methods for TSS identification, we measured the performance using accuracy, recall, FPR, FDR, and F1-score, which are defined as:

$$\mathrm{Accuracy}=\frac{\mathrm{TP}+TN}{\mathrm{TP}+TN+FP+FN}$$

$$\mathrm{Recall}=\frac{\mathrm{TP}}{\mathrm{TP}+FN}$$

$$\mathrm{FPR}=\frac{\mathrm{FP}}{\mathrm{FP}+TN}$$

$$\mathrm{FDR}=\frac{\mathrm{FP}}{\mathrm{FP}+TP}$$

$$F1\text{-}score=\frac{2TP}{2TP+FP+FN}$$

where TN means true negative and FN means false negative.

In binary classification, one region in our dataset can only be positive or negative and all metrics work without confusion. To better define the TP and FP during scanning along a certain region, we firstly searched for the strongest signal predicted by our model and counted signals with distance ≤ tolerant distance (100 bp) to the nearest real TSS as TP, otherwise FP. In addition, those real TSSs without a strong signal (> 0.5) as a neighbor were counted as FN.

### Clustering and empirical *P* value calculation

Since we randomly shifted the paired DNA sequence and coverage within 50 bp from the CAGE peak to allow a certain level of freedom for data augmentation during the training phase, the sites at the vicinity of true TSSs could also get relatively high prediction scores. Therefore, to reduce FDR by directly using outputs from the deep learning model, we developed a clustering-based method by grouping any sites with a prediction score (outputted from the model and the range is from 0 to 1) larger than 0.5 and within 10 bp into a cluster with merge function from BEDTools [22]. Any site with a prediction score below 0.5 was discarded during the clustering step. The details are shown in File S1D.

### Visualization of model features and identification of TFs

To understand sequence features learned by our model during the fine-tuning stage of meta-learning, we paired each filter in the convolution layer of the meta-model and the fine-tuned model of each cell type, and calculated the gain of motif information (GMI), defined as the difference of two positional weighted matrixes, among which each GMI was compared with all transcription factor (TF) binding profiles of *Homo sapiens* in JASPAR [23] to identify tissue-specific motifs and TFs using Tomtom from the MEME suite [24]. There were 64 filters for each cell type, and a total number of 640 filters (64 × 10) were obtained for 10 cell types, resulting in 640 GMIs. After matching 640 GMIs with the JASPAR database, the matched motif–TF pairs with *P* < 0.001 were selected and a total number of 157 unique TFs were identified. We further measured the expression levels of those TFs in 10 cell types. For each TF, we counted the profile from the fine-tuned models of 10 cell types (1: with an associated GMI; 0: without an associated GMI) and calculated the Pearson correlation between the profile and the expression level in 10 cell types. 42 TFs out of 157 TFs with concordant expression patterns (Pearson correlation coefficient > 0.2) were selected and visualized.

## Results

### A deep learning-based model for TSS prediction using both DNA sequence and RNA-seq coverage information

**Figure 1:**
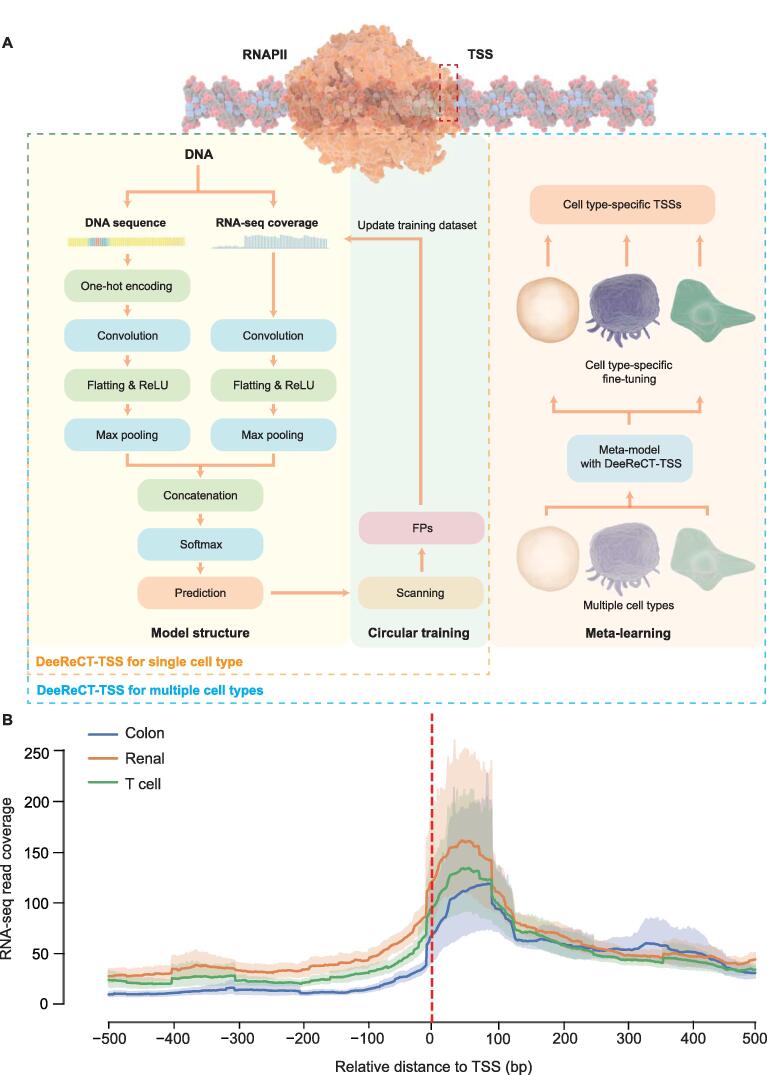
**A deep learning-based model for TSS prediction using both DNA sequence and RNA-seq coverage information** **A.** The schematic of the workflow and architecture of DeeReCT-TSS. DeeReCT-TSS uses both DNA sequence information from the reference genome and the corresponding coverage information from RNA-seq as inputs, and extracts features after a deep learning network for binary prediction of TSSs. During the training process of each epoch, we used the model for scanning and integrated the FPs obtained from scanning into the training dataset as new negative samples to continuously reduce the FPR of the model as circular training. For identifying cell type-specific TSSs in multiple cell types, we used a meta-learning mechanism to train the model with data from multiple cell types in order to obtain a consensus model that has a strong generalization ability across different cell types. Then, for a particular cell type, the model can be used to achieve better performance on the corresponding cell type by performing a one-step fine-tuning on the meta-model. By clustering the binary predictions from scanning the genome, we could obtain the final predicted TSS positions. Details of the model structure were described in Method. **B.** RNA-seq coverage is dramatically increased across the TSS. The dashed line indicates the position of TSS, the solid curve is the mean of RNA-seq converge across all true TSSs, and the shade is the 95% confidential interval of the coverage. RNA-seq, RNA sequencing; RNAPII, RNA polymerase II; ReLU, rectified linear unit; DeeReCT, Deep Regulatory Code and Tools; TSS; transcription start site; FP, false positive; FPR, false positive rate.

For TSS prediction, we built a deep learning model by taking both DNA sequence and RNA-seq coverage information as inputs. Both inputs went through two independent CNN models that had a convolution layer with 64 filters to extract motif features and the trend of coverage changes, respectively. Following the convolution layer, a ReLU was applied as the activation function followed by the max pooling layer. Two feature vectors were then concatenated together and fed into the FC layer. The last softmax layer gave the prediction value between 0 and 1. To improve the generalization capability, weight decay and dropout were applied (Figure 1A).

### Incorporating RNA-seq coverage information improves TSS prediction

To train our model, we downloaded the full set of annotated TSSs (n = 201,802) from FANTOM (https://fantom.gsc.riken.jp/5/), and then calculated their usage in three cell lines, including a colon carcinoma (COLO-320), a renal carcinoma (OS-RC-2), and a T cell leukemia cell lines (ATN-1), based on the respective CAGE-seq data (see Method; File S1A). The active TSSs from protein-coding genes were used as the positive dataset, with their corresponding DNA sequences and RNA-seq coverages as inputs. Then we randomly picked the same number of regions with a distance between 500 bp to 1000 bp from the nearest TSS peak as the initial negative datasets. Since we selected the activate TSSs as the positive dataset, the number of ground truth TSSs in different cell types was different. An average of ∼ 20,000 active TSSs from on average 11,000 protein-coding genes were obtained in each cell line (Table S1). By integrating RNA-seq data into the model, we could capture the change of the sequencing coverage around the TSS (Figure 1B). To compare our integrative model with that using only DNA sequence or RNA-seq coverage information alone, we did the ablation study, trained three models independently on the same training dataset, and tested the dataset with identical initial weights, biases, and number of epochs. Apparently, the integrative model achieves the best performance, which is on average 3% and 13% better than the sequence only model and coverage only model, respectively (Table S2). Overall, the integrated model takes information from both DNA sequences and RNA-seq coverages for TSS identification, resulting in the improvement of the performance compared to the model only using single source of the information.

### DeeReCT-TSS outperforms three state-of-the-art methods in the binary classification of promoters/TSSs

To further evaluate our method, we selected three state-of-the-art  methods on the promoter/TSS identification for the comparison, including PromID [15], iPSW(PseDNC-DL) [17], and TransPrise [16]. All these methods are based on CNNs, and the main difference between our method and the others is that DeeReCT-TSS is the only one that uses both DNA sequence and RNA-seq coverage information as inputs but the other three only use DNA sequence information. We trained each model with a very large number of epochs until (almost) convergence on the validation dataset. As shown in Table 1, our method outperforms all the three methods in terms of accuracy, recall, FDR, and F1-score. Since PromID is the one with the closest performance to our method and it is also the only published method that can control the FPs in a genome-wide scanning task, we only kept PromID for the following comparison in the genome-scale scanning task.

**Table 1:** **Comparison of the performance between DeeReCT-TSS and other three recently published methods in binary classification of TSSs on three datasets**

**Cell line**	**Metric**	**DeeReCT-TSS**	**PromID**	**TransPrise**	**iPSW(PseDNC-DL)**
Colon carcinoma cell line	Accuracy	0.93859	0.91653	0.90576	0.81711
	Recall	0.90855	0.89048	0.88549	0.72727
	FDR	0.03337	0.06057	0.07709	0.11341
	F1-score	0.93669	0.9143	0.90381	0.79906
Adult T cell leukemia cell line	Accuracy	0.90055	0.87174	0.82092	0.79513
	Recall	0.85616	0.83141	0.74811	0.7772
	FDR	0.06042	0.09563	0.12436	0.19389
	F1-score	0.89593	0.86635	0.80686	0.79139
Renal carcinoma cell line	Accuracy	0.91836	0.88151	0.85737	0.80165
	Recall	0.8518	0.8308	0.80262	0.70805
	FDR	0.01739	0.07542	0.09867	0.12886
	F1-score	0.91253	0.87518	0.84911	0.78117

*Note*: DeeReCT, Deep Regulatory Code and Tools; TSS, transcription start site; FDR, false discovery rate.

### DeeReCT-TSS enables genome-wide promoter/TSS identification by scanning the transcribed genome

Using the RNA-seq coverage information, we could reduce the number of sites for the genome-wide TSS scanning from 3 billion to ∼ 800 million, although even this reduced number of testing sites is well beyond the capacity of the previous TSS prediction methods to achieve a reasonable number of FPs. Since positive and negative datasets are highly unbalanced during the genome scanning, in which less than 0.01% of scanning sites are true TSSs and the rest are all negatives, we introduced an iterative negative data enhancement strategy into our model. In brief, we repeatedly trained the binary classification model by randomly substituting negative data in the training dataset with FPs predicted by the model trained in the previous round (Figure 1).

**Figure 2:**
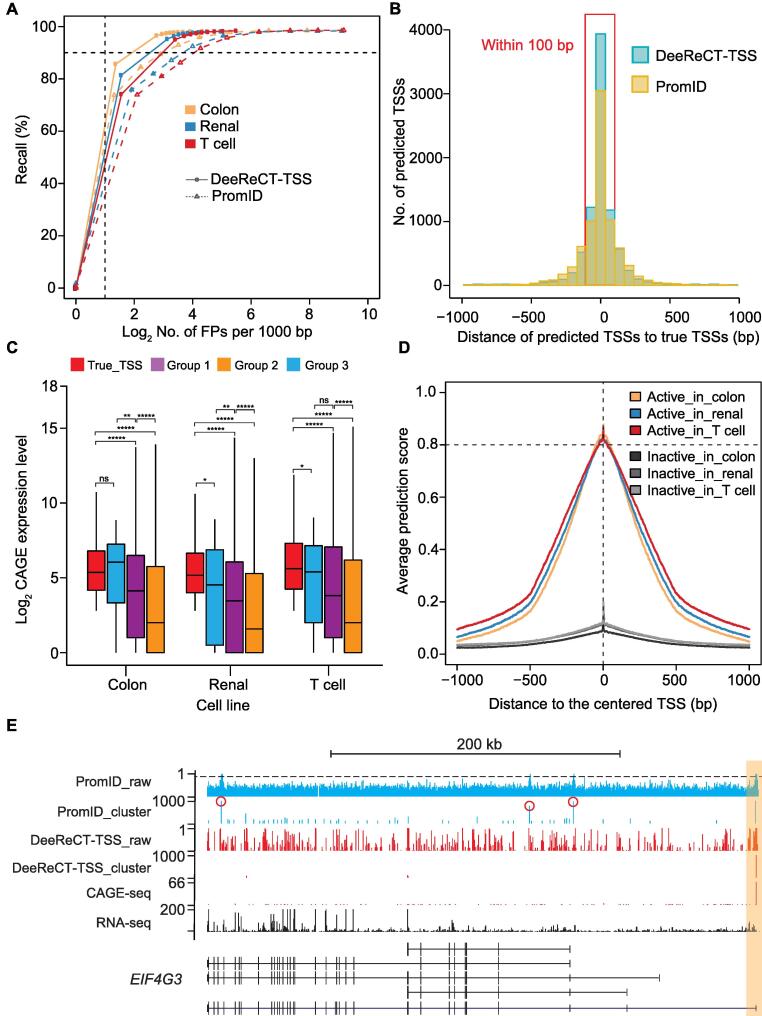
**DeeReCT-TSS outperforms a recently published method in TSS identification** **A.** The performance of DeeReCT-TSS and PromID on datasets from three cell lines. **B.** Histogram of the distance between predicted TSSs and true TSSs in the colon carcinoma cell line. **C.** Boxplot showing the CAGE expression of multiple groups of TSSs, including: Group 1 (TSSs predicted only by DeeReCT-TSS); Group 2 (TSSs predicted only by PromID); Group 3 (TSSs predicted by both DeeReCT-TSS and PromID); and true_TSS (ground truth TSSs in each cell line, which is the actively expressed annotated TSSs). *, *P* < 0.05; **, *P* < 0.01; *****, *P* < 1E–80; ns, not significant (Wilcox test). **D.** Meta-gene analysis of active TSSs and inactive TSSs with the prediction scores from DeeReCT-TSS in three cell lines. **E.** Genome browser view of CAGE-seq, RNA-seq, raw prediction scores outputted from DeeReCT-TSS (DeeReCT-TSS_raw) and PromID (PromID_raw), and scores after clustering (DeeReCT-TSS_cluster and PromID_cluster) for *EIF4G3*. TSSs falsely predicted by PromID were marked with red circles. CAGE, cap analysis of gene expression.

In the genome-wide scanning, we predicted TSS scores for 837,507,571, 892,712,017, and 760,462,470 sites covering 15,517, 15,982, and 15,736 protein-coding genes in the colon carcinoma, the renal carcinoma, and the T cell leukemia cell lines, respectively. As millions of negative sites were not predicted as TSSs (TN), while much fewer sites were predicted as TSSs (FP + TP), the FPR derived from FP divided by the sum of FP and TN is extremely low. Therefore, we defined a metric as the FPs in every 1000 bp to evaluate the performance of each method. In all of these three datasets, compared to PromID, our method could achieve a higher recall with a much lower number of FPs in every 1000 bp (Figure 2A). However, even though the FPRs of both methods are relatively low, due to a total number of ∼ 800 million query positions, millions of sites predicted as TSSs are still FPs. To address this, we further grouped the sites into clusters based on their prediction scores and used the score distribution in each cluster to evaluate whether it contains a true TSS or not (see Method). As we expected that sites in the vicinity of the true TSS should get high scores, the clusters harboring the true TSS should contain a very dense set of high score sites. In contrast, FPs could be derived from sparsely distributed high score sites. By taking this into account, we could transform predictions from ∼ 800 million sites to less than one million clusters.

In this way, we could reduce FDR dramatically, achieving ∼ 77% recall with ∼ 25% FDR in the colon carcinoma cell line (Figure S1A and B). Notably, the FDR is over estimated in our prediction as we found that around half of the FPs are still supported by the CAGE-seq, but their expression levels do not pass the threshold (Figure S1B; File S1E). We also investigated the potential causes of FNs. It turned out that the TSSs failed to be predicted (FN) usually have low expression levels, and weak TSS-related sequence features. The accurate prediction of these TSSs might need models to be trained by additional datasets or models incorporating additional features (Figure S1C and D; File S1E).

In comparison, after the clustering procedure, to get a 70% recall, FDR of the PromID method remains as high as 50% (Figure S1A). Moreover, the central positions of the clusters identified by our method are very close to true TSSs, which is much more accurate than PromID (Figure 2B, Figure S1E and F).

### DeeReCT-TSS could discriminate active TSSs from inactive TSSs

TSSs might be only used in certain cell types, and computational methods that are solely based on DNA sequences are unlikely to determine such cell type-specific TSS usage. In comparison, by incorporating RNA-seq information, our method should be able to address this issue. To assess the performance on this perspective, we split the predicted TSSs into three groups: the ones only predicted by our method (Group 1), the ones only predicted by PromID (Group 2), and the ones predicted by both methods (Group 3), and compared them with true TSSs in each cell line. By analyzing the expression levels of these TSSs in the respective cells, we found that the TSSs in Groups 1 and 3 were expressed based on CAGE-seq and the expression levels were only slightly lower than that of the true TSSs, whereas the TSSs only predicted by PromID (Group 2) were almost not expressed at all. Moreover, the expression of TSSs in Group 1 was significantly higher than that in Group 2, suggesting that a much higher proportion of TSSs predicted by our method are truly expressed compared to that predicted by PromID (Figure 2C).

We also performed an ablation study by only using DNA sequence information (the sequence only model) or the integration of RNA-seq data and DNA sequence information (the integrated model) to check the contribution of the RNA-seq data in TSS prediction with DeeReCT-TSS. Among 201,802 TSSs annotated in FANTOM, 17,725, 19,721, and 22,171 were used in colon, renal, and T cell with sufficient expression based on the CAGE-seq data and the rest were considered as unused. The integrated model detected slightly more used TSSs than the sequence only model in each cell line. More importantly, the number of FPs, including the unused TSSs and predictions without overlapping with any of the 201,802 TSSs in FANTOM, was much higher in the sequence only model than the integrated model (Table S3).

To further demonstrate that our method could distinguish active TSSs from inactive ones, we classified all TSSs as active or inactive based on their expression levels in each cell line. We then compared the predicted scores between these two groups of TSSs. As shown in Figure 2D, only active TSSs manifested the positive signals. As one example, 24 TSSs were annotated for *EIF4G3*, while only the proximal TSS was used in the colon carcinoma cell line (represented by CAGE-seq), which could be distinguished by our method (Figure 2E). In comparison, PromID predicted three more false TSSs, among which one is inactive annotated TSS and another two are even not annotated.

### Robustness of DeeReCT-TSS with different sequencing depths and the applications across multiple species

As DeeReCT-TSS requires RNA-seq data as one input, the sequencing depth of the RNA-seq data might impact its performance. To investigate this, we generated multiple datasets via subsampling of reads from RNA-seq data of T cell, as an example. These datasets represented different depths, including original (132 M), 100 M, 50 M, 10 M, and 5 M (where M stands for millions of sequencing reads). We then applied DeeReCT-TSS on each dataset and found that the performance was indeed affected by the sequencing depth. However, the identification of TSSs was quite robust once the sequencing depth was above 50 M (Table S4).

We also checked whether DeeReCT-TSS model trained in one species could be applied to another species. For this purpose, we applied DeeReCT-TSS on an RNA-seq data from mouse with a pre-trained model based on the data from human. As shown in Table S5, although the performance of the model on mouse was not as good as that of human, still, the model was able to detect more than half of the truly expressed TSSs. To be noted, unlike datasets from human that had the matched RNA-seq and CAGE-seq data from FANTOM in each cell type, RNA-seq and CAGE-seq data of mouse were from two independent sources. This might also introduce some batch effects for the predictions. Overall, these results showed high robustness of DeeReCT-TSS on RNA-seq data with reasonable sequencing depth and its transferability across multiple species.

### A meta-learning-based extension for predicting cell type-specific TSSs and associated TFs

**Figure 3:**
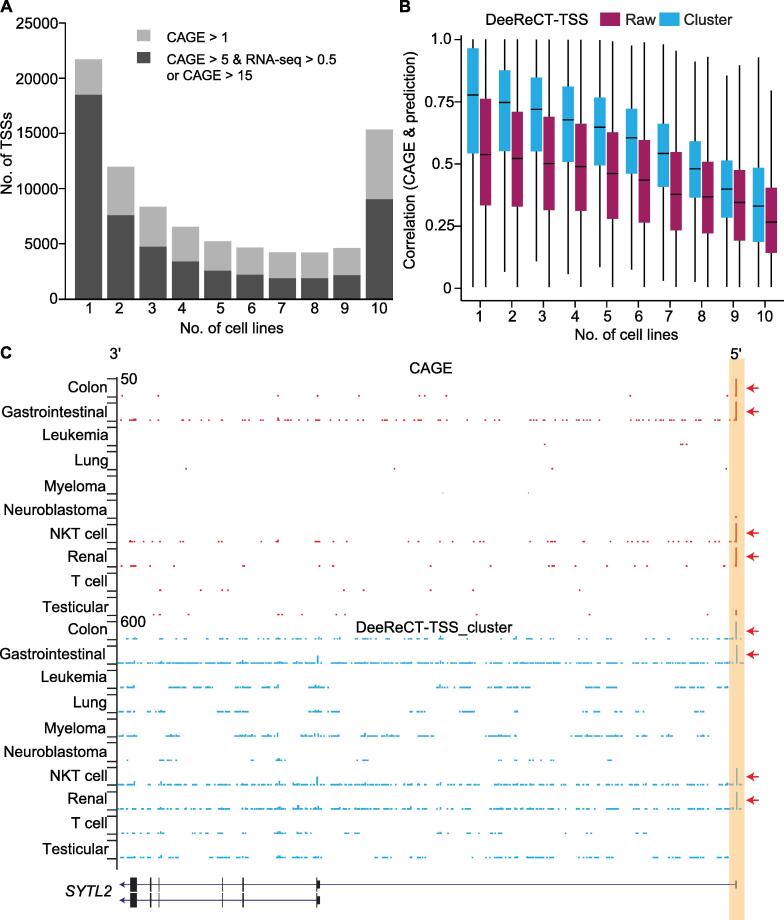
**DeeReCT-TSS is capable of identifying cell type-specific TSSs** **A.** Numbers of active TSSs in the corresponding number of cell lines. **B.** Boxplot illustrating correlations between prediction scores of TSSs from DeeReCT-TSS and CAGE expression across 10 cell lines. Each box indicating a group of TSSs expressed in corresponding cell line numbers. Raw represents the raw prediction score outputted from the model, and cluster represents the score after clustering of the raw score. **C.** UCSC genome browser visualization shows an example of TSS (highlighted in orange) from *SYTL2* that is active in the colon carcinoma, the gastrointestinal carcinoma, the NKT cell leukemia, and the renal carcinoma cell lines (represented by CAGE-seq), which was correctly predicted by DeeReCT-TSS. The red arrows indicate the cell lines, where the TSS is active or predicted. UCSC, University of California Santa Cruz; NKT, natural killer T.

Since DeeReCT-TSS is able to identify active TSSs in each cell type, we would like to further explore the commonality among different cell types and, at the same time, build a model that can be easily generalized to handle the difference among them. For this purpose, the state-of-the-art meta-learning strategy, which takes advantage of an abstract model trained with data from multiple tasks and is capable of good adaptation to a specific task with a mini fine-tuning session, exactly meets our needs. Here, the more specific aim was to train a generalized model across multiple cell types, which is also capable of being fine-tuned for any specific cell type. To check the feasibility of integrating the meta-learning strategy in DeeReCT-TSS, we extended our analysis to 10 cell lines and a total of 53,969 TSSs that are active in at least one cell type were used as the positive dataset. The number of TSSs that are active across different numbers of the cell lines shows a typical bimodal distribution, where the two peaks represent those only expressed in one cell line and those expressed in all 10 cell types, respectively (Figure 3A). Next, to obtain an abstract model with the maximum interpretability across all 10 cell types, we trained the meta-learning model with partial training data, whose proportion was required to be not large and was usually smaller than 50%, and then fine-tuned it to each cell type (see Method; Figure 1). We did the ablation study to show the performance of the fine-tuned model in 10 cell lines from meta-model trained using 5%, 10%, 20%, 30%, and 50% of TSSs in each cell line. Based on the F1-score, we found that the performance of the model using 20% of TSSs from each cell type for meta-training was almost saturated, while using 30% or 50% of TSSs had no significant effect to improve the model compared to the increasing effort to use larger proportional training data (Figure S2). Since we only took a small partial dataset (20%) from each cell type for training and used all remaining dataset for fine-tuning and testing, there was no need to further test our model on a complete new cancer type as in the traditional way. As shown in Table 2 and Table S2, on the binary classification task, the meta-model trained with 10 cell types decreased the FDR by 1% on average compared to the model trained solely on the colon carcinoma, the renal carcinoma, and the T cell leukemia cell lines, though with on average decreased F1-score by 4% as a trade-off for the generalized model. Based on the meta-model, the fine-tuned model shows better performance than both meta-model and the one trained solely in each cell line with on average 5% and 1% improvement on F1-score, respectively.

**Table 2:** **Performance of meta-model and fine-tuned model on 10 cell lines**

**DeeReCT-TSS**	**Metric**	**Recall**	**FDR**	**F1-score**
Meta-model	Natural killer T cell leukemia cell line	0.7273	0.03287	0.83024
	Colon carcinoma cell line	0.86235	0.02485	0.91529
	Gastrointestinal carcinoma cell line	0.7546	0.03171	0.84819
	Acute lymphoblastic leukemia cell line	0.71928	0.02943	0.82624
	Lung carcinoma cell line	0.74477	0.03022	0.84251
	Myeloma cell line	0.75222	0.02814	0.84805
	Neuroblastoma cell line	0.79193	0.02486	0.87403
	Renal carcinoma cell line	0.77725	0.02699	0.86418
	Adult T cell leukemia cell line	0.74834	0.03211	0.84407
	Testicular germ embryonal cell line	0.72749	0.03654	0.82901
Fine-tuned model	Natural killer T cell leukemia cell line	0.82357	0.05374	0.89206
	Colon carcinoma cell line	0.92227	0.04101	0.94027
	Gastrointestinal carcinoma cell line	0.86808	0.0531	0.90578
	Acute lymphoblastic leukemia cell line	0.85455	0.0653	0.89282
	Lung carcinoma cell line	0.86677	0.05926	0.90224
	Myeloma cell line	0.87594	0.05743	0.90803
	Neuroblastoma cell line	0.87728	0.04078	0.91641
	Renal carcinoma cell line	0.87969	0.04738	0.9147
	Adult T cell leukemia cell line	0.86289	0.05618	0.90154
	Testicular germ embryonal cell line	0.88168	0.07335	0.9036

We further applied the fine-tuned model on the genome scanning task in each cell line, and eventually, we identified TSSs with a recall around 73% and an FDR around 28%. Among these false predicted TSSs, half of them are annotated, but their expression levels do not pass the threshold, and only 10% of the total predicted TSSs are neither annotated nor supported by CAGE-seq (Figure S3). We also compared the results from the fine-tuned model to that from the model solely trained on the colon carcinoma cell line, which shows a better performance in terms of both recall (79.5% and 77.7%, respectively) and FDR (24.4% and 25.5%, respectively). To further evaluate the performance of our model to accurately predict the TSSs with specific expression patterns across 10 cell types, we correlated the prediction scores with the CAGE-seq-based expression levels across 10 cell lines for each TSS and obtained a strong positive correlation (median of Pearson correlation is 0.58). Notably, the correlation between prediction and CAGE-seq expression is the lowest (median of Pearson correlation is 0.24) for those TSSs that are active in all 10 cell lines, which is likely due to the fact that the prediction score can only determine if a TSS is active or not, but it is not a metric to measure the TSS expression level. In contrast, for those cell type-specific TSSs expressed in only one cell line, the median correlation coefficient is as high as 0.75 (Figure 3B). Figure 3C shows an example in *SYTL2*, which is active in the colon carcinoma, the gastrointestinal carcinoma, the natural killer T cell leukemia, and the renal carcinoma cell lines. Such usage pattern was successfully predicted by our method. Overall, these results suggest that our model could be generalized to identify active TSSs in different cell types.

**Figure 4:**
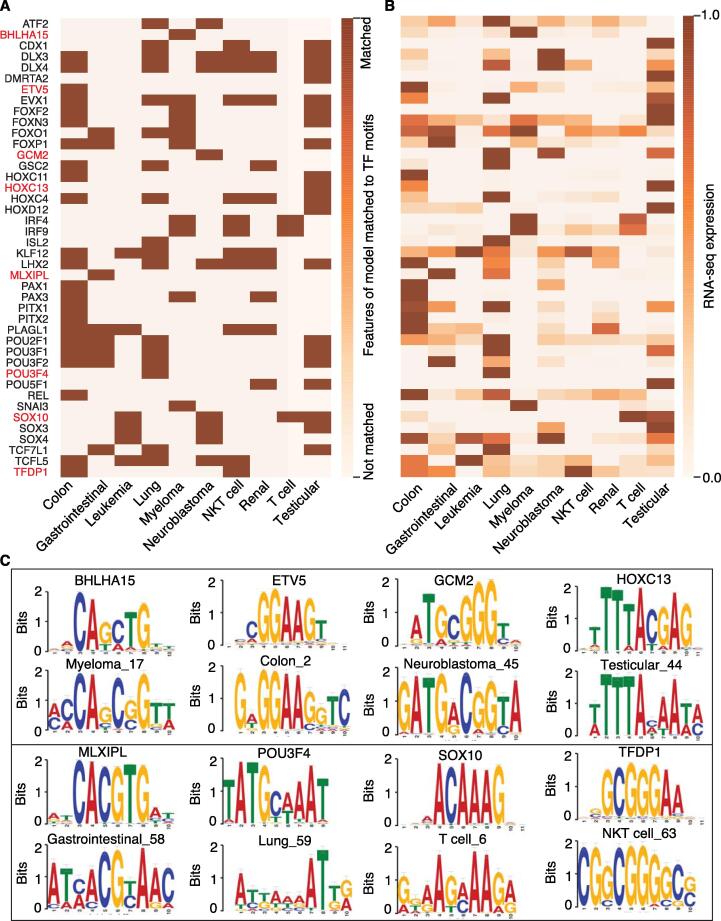
**Identification of putative *cis*-elements around TSSsand the corresponding TFs across 10 cell lines** **A.** Heatmap showing that the identified TFs matched with sequence elements extracted from the deep learning model in each cell line. **B.** Heatmap showing the normalized expression level of the TFs across 10 cell lines. **C.** Examples showing the binding motifs of the identified TFs and sequence elements extracted from the deep learning model from different cell lines (marked in red in A). TF, transcription factor.

One major advantage of our meta-learning model is that its abstract model can capture common features of TSSs among all cell lines, while the fine-tuned model to each cell line has a preference to identify the active TSSs in the corresponding cell line by enhancing the usage of the cell type-specific features. To this end, we visualized the GMI from the convolutional layers by comparing the difference between paired filters of the meta-model and the fine-tuned model of each cell type and matched those GMI to the motifs of TF-binding sites (see Method). In total, 42 putative TFs with concordant expression patterns (Pearson correlation coefficient ≥ 0.5) were identified across 10 cell lines (Figure 4A and B). These TFs that are specifically expressed in some cell types, potentially regulate the corresponding cell type-specific TSSs. For example, BHLHA15/MIST1 identified in myeloma has been reported as a plasmacytic differentiation marker and potentially controlled transcriptional network with stage-specific overexpression during plasma cells differentiation [25], [26]. ETV5 identified in colon carcinoma cell lines has been reported to be abnormally upregulated in the colorectal cancer and positively correlated with the tumor size, lymphatic metastasis, tumor node metastasis, and worse survival [27]. Examples of the TF motifs and the matched *cis*-elements extracted from deep learning model in different cell lines are shown in Figure 4C.

### The trained meta-learning model could be applied to other independent datasets

**Figure 5:**
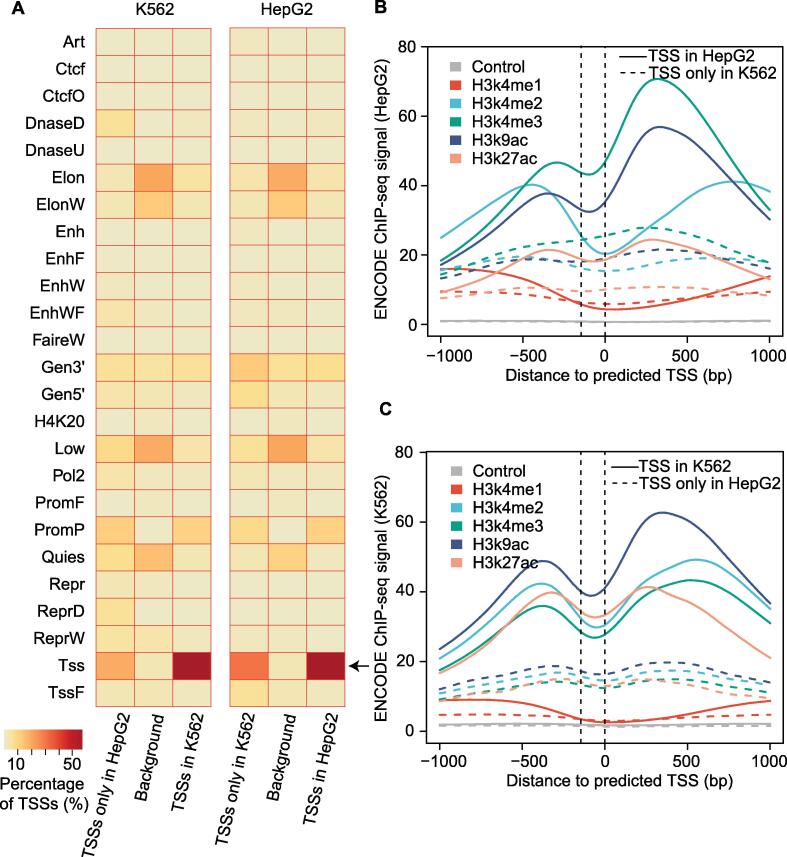
**TSSs predicted in K562 and HepG2 by DeeReCT-TSS could be validated by chromatin states identified from epigenetic information** **A.** Heatmap showing the proportion of predicted TSSs located in each chromatin state in K562 and HepG2. The background was generated by randomly selecting regions, which were covered by RNA-seq data. **B.** Meta-gene analysis shows that the predicted TSSs could be located within the NDR in the promoter region in HepG2. **C.** Meta-gene analysis shows that the predicted TSSs could be located within the NDR in the promoter region in K562. Putative NDR within the promoter was indicated by two vertical dashed lines. NDR, nucleosome-depleted region; ENCODE, the ENCyclopedia Of DNA Element; ChIP-seq, chromatin immunoprecipitation followed by high-throughput sequencing.

As the information for the active TSSs could also be encoded at the chromatin level, to further evaluate the generalization ability on TSS identification of our meta-learning extended model to unseen datasets, we applied it to two cell lines HepG2 and K562, for which ENCODE project collected both RNA-seq and different histone modification data. In total, we predicted 14,569 and 13,465 putative TSSs in HepG2 and K562, respectively. Next, we associated the prediction with chromatin states of these two cell lines, which were identified by using a series of histone modification data and TF ChIP-seq data in the ENCODE project, including Tss, TSS flanking region (TssF), and PromP (inactive promoter). The results showed that our predictions were highly consistent with Tss chromatin state in these two cell lines. Importantly, the overlap between the predicted TSSs in HepG2 and the Tss chromatin state from K562, as well as the overlap between the predicted TSSs in K562 with the Tss chromatin state from HepG2, were much lower than that within the same cell type, suggesting the high cell type specificity of our prediction (Figure 5A).

As regions of the ENCODE chromatin state are still very wide (∼ 500 bp), to inspect our predicted TSS position more precisely, we performed the meta-gene analysis of the predicted TSSs with H3K4m1, H3K4m2, H3K4m3, H3K9ac, and H3K27ac ChIP-seq signals in single base-pair resolution. Surprisingly, our predicted TSSs were exactly located within the NDR flanked by the modified histones, where the transcription was likely initiated. Again, this pattern could only be observed by overlapping results from the same cell line (Figure 5B and C), demonstrating again a high accuracy and specificity of our method. Taken together, all these data suggest that DeeReCT-TSS is a novel computational method that is capable of accurately predicting active TSSs in a genome-wide manner. It could be widely used to study TSS usage across multiple cell types or tissues, as well as between different pathophysiology conditions as long as conventional RNA-seq data are available.

## Discussion

Computational methods for TSS prediction have been used to annotate TSSs and to study regulatory mechanisms via *cis-*elements residing around the TSS. One shortage limiting their practical applications is that the majority of them can only do binary classification between sequences with TSSs and the ones without TSSs, on a balanced dataset. However, in the human genome, these positive and negative sequences are highly unbalanced, while the number of negative sequences is 15,000 times more than the positive ones. In DeeReCT-TSS, we addressed this challenge by several means. First, we iteratively enhanced the negative dataset, which dramatically decreases the amount of potential FPs by forcing the model to distinguish between active TSSs and inactive TSSs with similar features. In addition, we incorporated RNA-seq data information, which significantly improves the accuracy of the predicted TSS positions. Also, as we filtered out a huge number of query positions, this also helped to decrease the FDR when performing the genome scanning task. Moreover, we introduced a clustering-based method on the prediction scores from the model, which further greatly reduces the FDR. Finally, meta-learning technology worked together with those strategies to ensure the robust and generalizable performance of DeeReCT-TSS in the genome-scale TSS identification task.

In spite of the fact that the DNA sequence of a promoter is identical in different cell types and tissues, their transcription profiles are highly variable, resulting in distinct landscapes of active TSSs. In the last decades, the FANTOM project annotated more than 200,000 TSSs in the human genome by combining CAGE-seq data from dozens of cell types, while only a handful of them (∼ 10%) could be active in a single cell type or tissue. Discriminating whether a TSS is active or inactive in each cell type or tissue could be equally important as predicting a novel TSS in studying gene regulation across cell types/tissues and between different physiology conditions. To our knowledge, DeeReCT-TSS is the only computational method that is capable of accurately identifying active TSSs in a cell type by incorporating RNA-seq data and meta-learning. By applying our method, we expect to identify more than 70% of the active TSSs and about 10% of our predicted TSSs might be true FPs. As our method takes both DNA sequence and RNA-seq coverage as inputs, some issues of RNA-seq, such as genes with low expression and misalignment of the RNA-seq reads, might affect the performance of our method. Indeed, we found that our method is more powerful to identify TSSs from genes with higher expression compared to that from genes with lower expression (Figure S1D). This could be one limitation of our method, while it might be neglected in practical studies when comparing two groups of samples by removing candidates showing significant differential expression. Even though there might be some misalignment of RNA-seq reads due to uniqueness, our model might be able to handle this as it also takes DNA sequence as inputs. On the other hand, for the species that have been extensively studied, such as human and mice, the misalignment of reads to the reference with the well-established tools could be very rare and its impact on our method could be very minor.

DeeReCT-TSS also has some limitations in comprehensively identifying TSSs. Since more than 90% of the human genome are non-coding and many non-coding genes may play important roles in gene regulation, the feasibility of applying DeeReCT-TSS on detecting TSSs for non-coding genes remains need to be explored. It might be used for several species of non-coding genes and fail for the rest, depending on the characteristics of non-coding genes, such as the expression level and whether they are independent transcriptional units. In addition, DeeReCT-TSS was trained by using the data from FANTOM5, which has the matched data (RNA-seq and CAGE-seq) for different cell lines but without data for histone modifications and chromatin states. Further integrating epigenetic markers could potentially improve the performance as more information would be taken for TSS prediction. However, to date it is very challenging to find available datasets with these three types of sequencing data simultaneously. Still, we also plan to further improve DeeReCT-TSS and take more sources of information into consideration in the future version, once we can get high-quality data with RNA-seq, CAGE-seq, and CHIP-seq simultaneously.

Finally, as demonstrated in Figure 5, the meta-model trained from the 10 cell lines, could be generalized to other independent samples with only conventional RNA-seq data (HepG2 and K562). Therefore, our model is capable of being applied to characterize active TSSs in many other cell types by using the corresponding RNA-seq data. Recently, a pan-cancer analysis using thousands of RNA-seq samples from The Cancer Genome Atlas (TCGA) project revealed the widespread alternative promoter regulations [28]. However, it only studied promoters from annotated TSSs and quantified promoter activity using splicing junctions of the first exon. On one hand, there might be a tremendous amount of unannotated TSSs expressed in cancers given that they often showed high abnormal transcriptional activities. On the other hand, the first splicing junctions could be hundreds and thousands bp away from TSSs, which limited the spatial resolution of that study in finding the change of TSS positions. We believe that DeeReCT-TSS could solve these problems and it could be used to identify TSSs that are specifically active in each cancer type by applying it on the TCGA RNA-seq datasets.

## Code availability

DeeReCT-TSS, which is implemented as an python software and accompanied by a user guide, is available at https://github.com/JoshuaChou2018/DeeReCT-TSS_release and https://ngdc.cncb.ac.cn/biocode/tools/BT007316.

## CRediT author statement

**Juexiao Zhou:** Conceptualization, Methodology, Formal analysis, Software, Validation, Data curation, Visualization, Writing - original draft, Writing - review & editing. **Bin Zhang:** Conceptualization, Methodology, Formal analysis, Software, Validation, Data curation, Visualization, Writing - original draft, Writing - review & editing. **Haoyang Li:** Formal analysis, Writing - review & editing. **Longxi Zhou:** Formal analysis, Writing - review & editing. **Zhongxiao Li:** Formal analysis, Writing - review & editing. **Yongkang Long:** Formal analysis, Writing - review & editing. **Wenkai Han:** Writing - review & editing. **Mengran Wang:** Validation, Writing - review & editing. **Huanhuan Cui:** Writing - review & editing. **Jingjing Li:** Software, Validation. **Wei Chen:** Conceptualization, Investigation, Writing - review & editing, Supervision, Project administration, Funding acquisition. **Xin Gao:** Conceptualization, Investigation, Writing - review & editing, Supervision, Project administration, Funding acquisition. All authors have read and approved the final manuscript.

## Competing interests

The authors have declared no competing interests.

## Supplementary material

Supplementary File S1 **Supplementary method**

Supplementary Figure S1 **DeeReCT-TSS outperforms a recently published method in TSS identification A.** Performance of DeeReCT-TSS and PromID in genome scanning after clustering the prediction score. **B.** Barplot showing the number of four groups of false predicted TSSs, including: anno & CAGE-seq > 0, which is the TSSs annotated in FANTOM and supported by CAGE-seq, but the expression did not pass the threshold; anno & CAGE-seq = 0, which is the TSSs annotated in FANTOM but not supported by CAGE-seq; unanno & CAGE-seq > 0, which is the TSSs not annotated in FANTOM, but supported by CAGE-seq; unanno & CAGE-seq = 0, which is the TSSs not annotated in FANTOM and not supported by CAGE-seq. Their percentages among the total predicted TSSs in each cell line were labeled in the bar. **C.** Cumulative plot showing the distribution of prediction score in binary classification for TSSs that are still successfully predicted in genome scanning and not in the three cell lines. **D.** Expression level of TSSs that are successfully predicted in genome scanning and not in the three cell lines. **E.** Histogram of distance between predicted TSSs and true TSSs in renal. **F.** Histogram of distance between predicted TSSs and true TSSs in T cell. FANTOM, Functional Annotation of The Mammalian Genome.

Supplementary Figure S2 **Ablation study showing performance of the fine-tuned model in 10 cell lines from meta-model trained using 5%, 10%, 20%, 30%, and 50% of TSSs in each cell line** X-axis is the proportion of TSSs used for meta-model in each cell line, and Y-axis is the average F1-score of the fine-tuned model. 10 cell lines include: a colon carcinoma cell line (COLO-320), a gastrointestinal carcinoma cell line (ECC12), an acute lymphoblastic leukemia cell line (HPB-ALL), a small cell lung carcinoma cell line (NCI-H82), a myeloma cell line (PCM6), a neuroblastoma cell line (CHP-134), a NKT cell leukemia cell line (KHYG-1), a renal carcinoma cell line (OS-RC-2), an adult T cell leukemia cell line (ATN-1), and a testicular germ cell embryonal carcinoma cell line (NEC15).

Supplementary Figure S3 **DeeReCT-TSS is capable of predicting cell type-specific TSSs across 10 cell types** Barplot showing the percentage of four groups of false predicted TSSs. Four groups are the same as Figure S1B. The line above the bar indicates the recall of the prediction in each cell line, while the dashed line indicates 70%.

Supplementary Table S1 **The number of TSSs and the corresponding genes from each cell line used in this study**

Supplementary Table S2 **Performance of deep learning-based model on TSS binary classification**

Supplementary Table S3 **Performance of DeeReCT-TSS on genome-wide TSSs scanning**

Supplementary Table S4 **Performance of TSSs prediction on five T cell datasets with different sequencing depths**

Supplementary Table S5 **Performance TSSs prediction on mouse dataset by applying pre-trained DeeReCT-TSS model based on human dataset**

## References

[1] Danino Y.M., Even D., Ideses D., Juven-Gershon T. (2015). The core promoter: at the heart of gene expression. Biochim Biophys Acta.

[2] Konoshita T., Makino Y., Wakahara S., Ido K., Yoshida M., Kawai Y. (2004). Candidate *cis*-elements for human renin gene expression in the promoter region. J Cell Biochem.

[3] Triska M., Ivliev A., Nikolsky Y., Tatarinova T.V. (2017). Analysis of *cis*-regulatory elements in gene co-expression networks in cancer. Methods Mol Biol.

[4] Shiraki T., Kondo S., Katayama S., Waki K., Kasukawa T., Kawaji H. (2003). Cap analysis gene expression for high-throughput analysis of transcriptional starting point and identification of promoter usage. Proc Natl Acad Sci U S A.

[5] Forrest A.R.R., Kawaji H., Rehli M., Baillie J.K., de Hoon M.J.L., Haberle V. (2014). A promoter-level mammalian expression atlas. Nature.

[6] Barth T.K., Imhof A. (2010). Fast signals and slow marks: the dynamics of histone modifications. Trends Biochem Sci.

[7] Ernst J., Kellis M. (2012). ChromHMM: automating chromatin-state discovery and characterization. Nat Methods.

[8] Solovyev V., Salamov A. (1997). The Gene-Finder computer tools for analysis of human and model organisms genome sequences. Proc Int Conf Intell Syst Mol Biol.

[9] Wingender E. (1994). Recognition of regulatory regions in genomic sequences. J Biotechnol.

[10] Solovyev V.V., Shahmuradov I.A. (2003). PromH: promoters identification using orthologous genomic sequences. Nucleic Acids Res.

[11] Knudsen S. (1999). Promoter2.0: for the recognition of PolII promoter sequences. Bioinformatics.

[12] Bajic V.B., Seah S.H. (2003). Dragon Gene Start Finder: an advanced system for finding approximate locations of the start of gene transcriptional units. Genome Res.

[13] Umarov R.K., Solovyev V.V. (2017). Recognition of prokaryotic and eukaryotic promoters using convolutional deep learning neural networks. PLoS One.

[14] Shahmuradov I.A., Umarov R.K., Solovyev V.V. (2017). TSSPlant: a new tool for prediction of plant Pol II promoters. Nucleic Acids Res.

[15] Umarov R., Kuwahara H., Li Y., Gao X., Solovyev V. (2019). Promoter analysis and prediction in the human genome using sequence-based deep learning models. Bioinformatics.

[16] Pachganov S., Murtazalieva K., Zarubin A., Sokolov D., Chartier D.R., Tatarinova T.V. (2019). TransPrise: a novel machine learning approach for eukaryotic promoter prediction. PeerJ.

[17] Tayara H., Tahir M., Chong K.T. (2020). Identification of prokaryotic promoters and their strength by integrating heterogeneous features. Genomics.

[18] Xia Z., Li Y., Zhang B., Li Z., Hu Y., Chen W. (2019). DeeReCT-PolyA: a robust and generic deep learning method for PAS identification. Bioinformatics.

[19] Li Z., Li Y., Zhang B., Li Y., Long Y., Zhou J. (2022;20:483–495). DeeReCT-APA: prediction of alternative polyadenylation site usage through deep learning. Genomics Proteomics Bioinformatics.

[20] Li H., Handsaker B., Wysoker A., Fennell T., Ruan J., Homer N. (2009). The Sequence Alignment/Map format and SAMtools. Bioinformatics.

[21] Nichol A, Achiam J, Schulman J. On first-order meta-learning algorithms. arXiv 2018;1803.02999.

[22] Quinlan A.R., Hall I.M. (2010). BEDTools: a flexible suite of utilities for comparing genomic features. Bioinformatics.

[23] Fornes O., Castro-Mondragon J.A., Khan A., van der Lee R., Zhang X., Richmond P.A. (2020). JASPAR 2020: update of the open-access database of transcription factor binding profiles. Nucleic Acids Res.

[24] Bailey T.L., Johnson J., Grant C.E., Noble W.S. (2015). The MEME suite. Nucleic Acids Res.

[25] Kassambara A., Herviou L., Ovejero S., Jourdan M., Thibaut C., Vikova V. (2021). RNA-sequencing data-driven dissection of human plasma cell differentiation reveals new potential transcription regulators. Leukemia.

[26] Yeung C.C.S., Mills J.C., Hassan A., Kreisel F.H., Nguyen T.T., Frater J.L. (2012). MIST1-a novel marker of plasmacytic differentiation. Appl Immunohistochem Mol Morphol.

[27] Cheng X., Jin Z., Ji X., Shen X., Feng H., Morgenlander W. (2019). ETS variant 5 promotes colorectal cancer angiogenesis by targeting platelet-derived growth factor BB. Int J Cancer.

[28] Demircioğlu D., Cukuroglu E., Kindermans M., Nandi T., Calabrese C., Fonseca N.A. (2019). A pan-cancer transcriptome analysis reveals pervasive regulation through alternative promoters. Cell.

