# Supplementary material for: Annotating TSSs in Multiple Cell Types Based on DNA Sequence and RNA-seq Data via DeeReCT-TSS
Source: Genomics Proteomics Bioinformatics. 2022 Dec 15;20(5):959–73. doi: 10.1016/j.gpb.2022.11.010 (PMC10025762; doi:10.1016/j.gpb.2022.11.010)
Supplement: Supplementary File S1 — Supplementary method [file mmc1.docx]

**File S1 Supplementary method**

1. **Data preparation for deep neural networks**

Our model has two inputs, the DNA sequence and the read coverage from RNA sequencing (RNA-seq) data. DNA sequences were extracted from the human reference genome (hg19), and coverages were calculated based on RNA-seq bam files with BEDTools and SAMtools from the Functional Annotation of The Mammalian Genome (FANTOM) (<https://fantom.gsc.riken.jp/5/>). We firstly selected 3 cell lines to train our model, including the renal carcinoma cell line, the adult T cell leukemia cell line, and the colon carcinoma cell line. To obtain the active transcription start sites (TSSs) in each cell line, we downloaded the robust cap analysis of gene expression (CAGE) peaks for human samples from FANTOM, which consists of 201,801 peaks. Next, for each cell line, we calculated the expression of each peak based on the downloaded homogeneous hCAGE (CAGE sequencing on HeliScope^TM^ Single Molecule Sequencer) data and RNA-seq data from FANTOM. The expressed TSSs were defined by requiring CAGE-seq score > 5 and RNA-seq coverage > 0.5 reads per million (RPM), or CAGE-seq score > 15. Finally, we only kept those TSSs located in regions (from upstream 5kb of the gene start to the gene end) of protein-coding genes.

To obtain the inputs for training, we calculated the coverage at each genomic site based on RNA-seq bam files. For sites within each active TSS peak, we extracted the DNA sequences and RNA-seq coverages from -500 bp to 501 bp as the positive dataset. Then we randomly picked the same number of regions with a distance between 500 bp to 1000 bp from the nearest TSS peak as the initial negative dataset. The full dataset was split into the training dataset (90%) and the test dataset (10%), and in total 5 random splits were performed, and the average performance was reported.

The regions for genome scanning were defined by RNA-seq data. We extracted RNA-seq coverage at each genomic site and merged any sites covered by more than one RNA-seq read within 1000 bp using BEDTools.

1. **The algorithm for circular training**

| **Algorithm 1** Circular training for reducing false positive |
| --- |
| Initialize $M$ (the model of DeeReCT-TSS), $D$ (the training dataset), and $V$ (the test dataset)  **for** iteration $=1, 2, 3, \ldots$ **do**  Model training on $D$ and select the best model $M^{*}$ with the highest F1-score on $V$  Scan for false positives on extended regions of $D$  Randomly replace half of negatives in $D$ with false positives above  **end for** |

1. **The algorithm for meta-training**

| **Algorithm 2** Meta-learning for DeeReCT-TSS |
| --- |
| Initialize $\phi$, the vector of the initial parameter  **for** iteration $=1, 2, 3, \ldots$ **do**  **for** each cell line (task) $\tau$ of all cell lines (tasks) $T$ **do**  Sample data of cell line (task) $\tau$, corresponding to loss $L_{\tau}$ on weight vectors $\tilde{\phi}$  Compute $\tilde{\phi}=U_{\tau}^{k}(\phi)$, denoting $k$ steps of gradients descent  Update $\phi\leftarrow\phi+\epsilon(\tilde{\phi}-\phi)$  **end for**  **end for** |

1. **The clustering-based method for deep learning output**

The final score of each cluster was calculated by summing up prediction scores at each site within this cluster. Suppose that we have a cluster $c$ that includes $n$ sites, the final score $S_{c}$ was calculated based on the formula below.

$$S_{c}=\sum_{i=1}^{n} S_{i}$$

where $S_{i}$ is the prediction score outputted from the model in each site, and $S_{i}$ will be assigned to 0 if it is smaller than 0.5.

To further estimate an empirical *P* value for each cluster, we made a null hypothesis that there was not any true TSS within the cluster, while the alternative hypothesis was that the cluster contained true TSS. The probability to reject the null hypothesis and accept alternative hypothesis is known as *P* value, which was roughly calculated as shown below. The probability $P\left( S_{i} \right)$ of a site $i$ with score $S_{i}$to be a true TSS was calculated by the total number of true TSSs with score above $S_{i}$ divided by the total number of TSSs with score above $S_{i}$ in the prediction. For instance, probability of a site with the prediction score 0.1 to be a true TSS, is 2.9% among all sites with score above 0.1 from the three cell lines. For a cluster with $n$ sites, the probability ($P_{c}$) that it does not contain any true TSS was calculated below.

$$P_{c}=\prod_{i=1}^{n} (1- P\left( S_{i} \right))\leq\prod_{i=1}^{int(S_{c})} (1-0.029)$$

The $P\left( S_{i} \right)$ is the probability of a site with score $S_{i}$ that is not a true TSS and $int\left( S_{c} \right)$ is the round number of $S_{c}$. Notably, a higher $S_{i}$ value will get a smaller $P\left( S_{i} \right)$, meanwhile $n$ should be always no less than $int\left( S_{c} \right)$.

$$P value=P_{c} = \prod_{i=1}^{int(S_{c})} (1-0.029)$$

To simplify the calculation for each cluster, as well as to link the *P* value to the final prediction score $S_{c}$, we used the maximum probability as the *P* value.

1. **Analysis of false predicted TSS by DeeReCT-TSS**

Notably, among the false positive predictions (28.8% of the total predicted TSSs), 9.3% are still annotated in FANTOM and supported by CAGE-seq, but their expression levels do not pass our expression threshold. 4.0% are annotated but not supported by CAGE-seq and 3.8% are not annotated in FANTOM but supported by CAGE-seq. Only ~ 11.7% of the total predicted TSSs are unannotated and not supported by CAGE-seq (Figure S1B). For the false negatives, by comparing the TSS prediction in binary classification and genome scanning, we found that the sites still predicted as true TSSs in genome scanning had a higher binary classification scores than those were not identified in the genome scanning, suggesting TSSs with moderate scores in binary classification could be dropped during circular training (Figure S1C). Moreover, the TSSs with higher expression level were more likely to be successfully predicted in genome scanning than those with lower expression (Figure S1D).
