# Supplementary figures and images for: Annotating TSSs in Multiple Cell Types Based on DNA Sequence and RNA-seq Data via DeeReCT-TSS
Source: Genomics Proteomics Bioinformatics. 2022 Dec 15;20(5):959–73. doi: 10.1016/j.gpb.2022.11.010 (PMC10025762; doi:10.1016/j.gpb.2022.11.010)

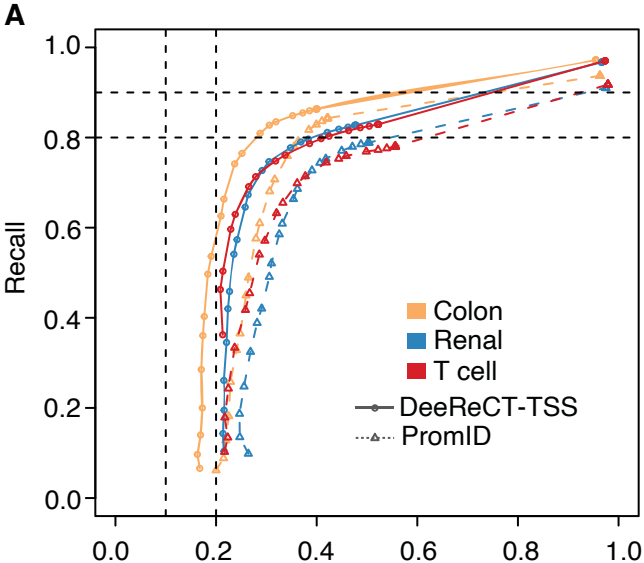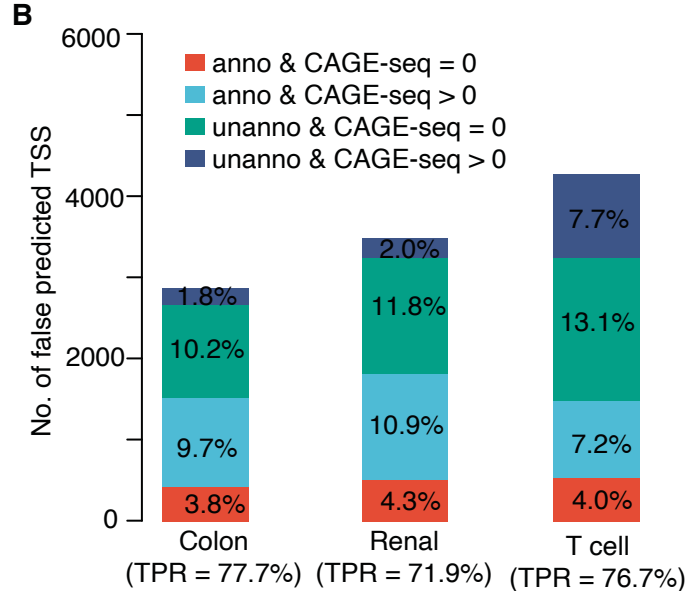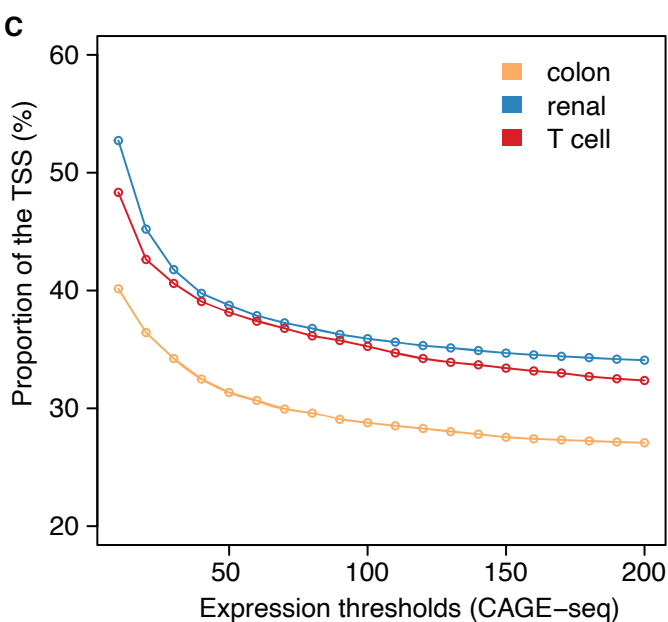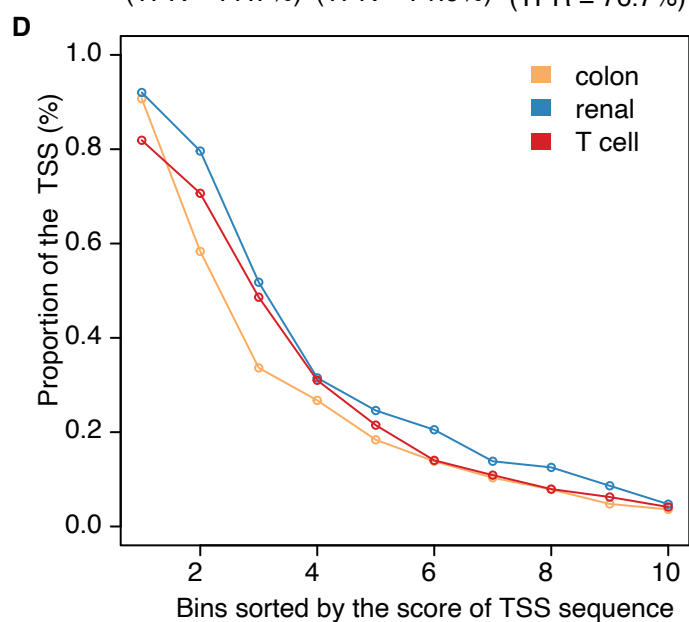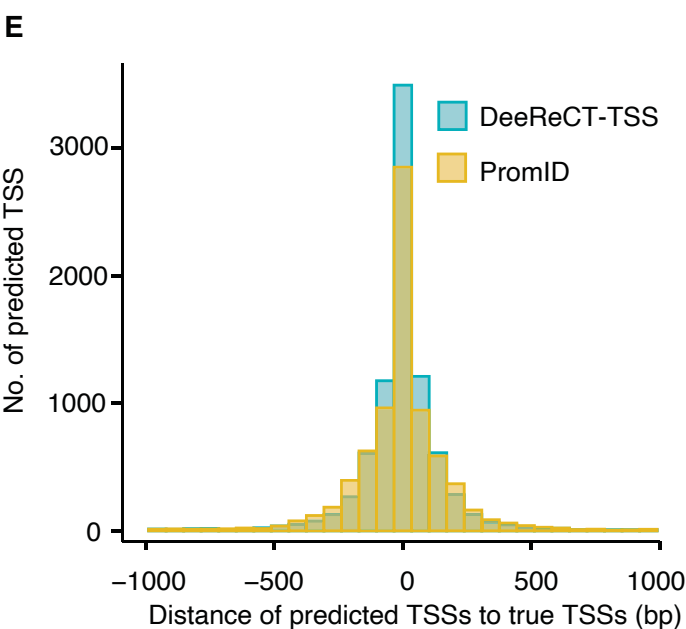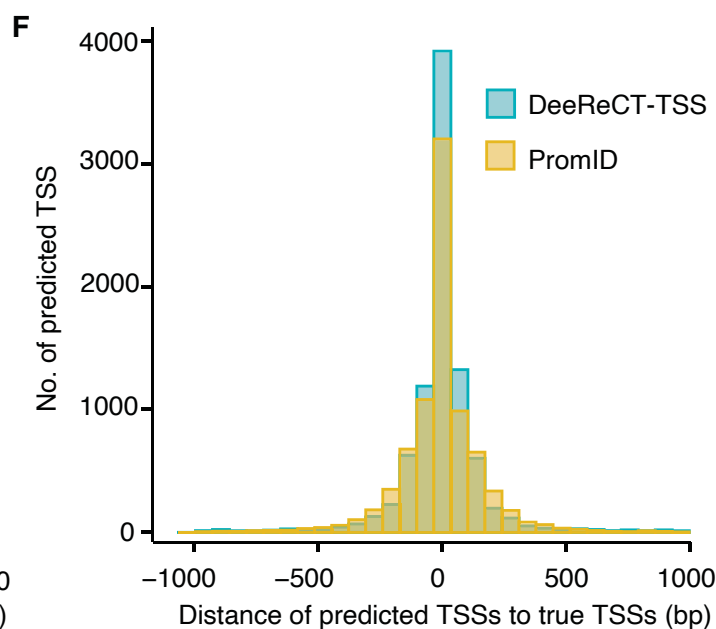

Supplement: Supplementary Figure S1 — DeeReCT-TSS outperforms a recently published method in TSS identification A. Performance of DeeReCT-TSS and PromID in genome scanning after clustering the prediction score. B. Barplot showing the number of four groups of false predicted TSSs, including: anno & CAGE-seq > 0, which is the TSSs annotated in FANTOM and supported by CAGE-seq, but the expression did not pass the threshold; anno & CAGE-seq = 0, which is the TSSs annotated in FANTOM but not supported by CAGE-seq; unanno & CAGE-seq > 0, which is the TSSs not annotated in FANTOM, but supported by CAGE-seq; unanno & CAGE-seq = 0, which is the TSSs not annotated in FANTOM and not supported by CAGE-seq. Their percentages among the total predicted TSSs in each cell line were labeled in the bar. C. Cumulative plot showing the distribution of prediction score in binary classification for TSSs that are still successfully predicted in genome scanning and not in the three cell lines. D. Expression level of TSSs that are successfully predicted in genome scanning and not in the three cell lines. E. Histogram of distance between predicted TSSs and true TSSs in renal. F. Histogram of distance between predicted TSSs and true TSSs in T cell. FANTOM, Functional Annotation of The Mammalian Genome. [file mmc2.pdf]

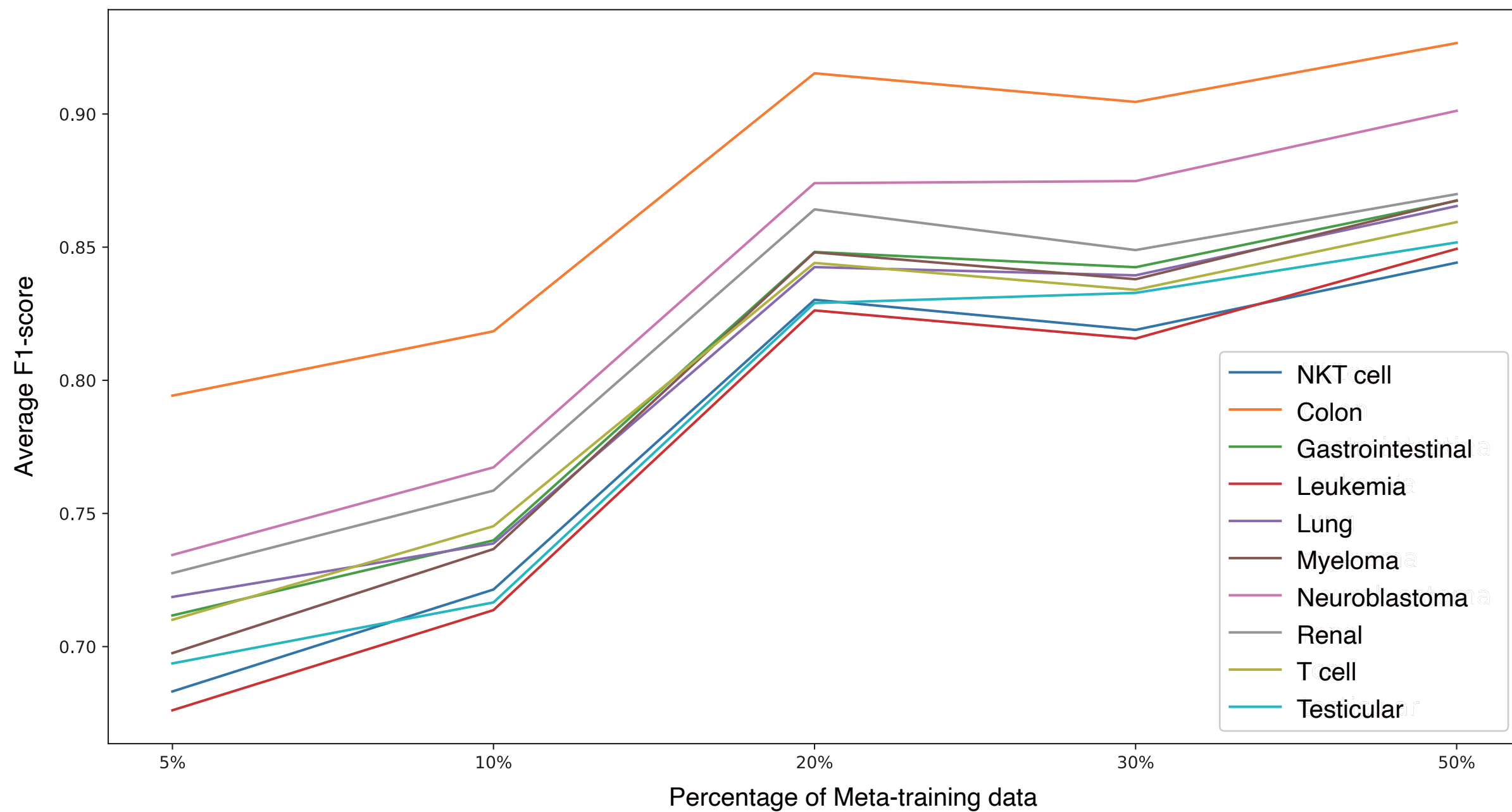

Supplement: Supplementary Figure S2 — Ablation study showing performance of the fine-tuned model in 10 cell lines from meta-model trained using 5%, 10%, 20%, 30%, and 50% of TSSs in each cell line X-axis is the proportion of TSSs used for meta-model in each cell line, and Y-axis is the average F1-score of the fine-tuned model. 10 cell lines include: a colon carcinoma cell line (COLO-320), a gastrointestinal carcinoma cell line (ECC12), an acute lymphoblastic leukemia cell line (HPB-ALL), a small cell lung carcinoma cell line (NCI-H82), a myeloma cell line (PCM6), a neuroblastoma cell line (CHP-134), a NKT cell leukemia cell line (KHYG-1), a renal carcinoma cell line (OS-RC-2), an adult T cell leukemia cell line (ATN-1), and a testicular germ cell embryonal carcinoma cell line (NEC15). [file mmc3.pdf]

% of predicted TSSs

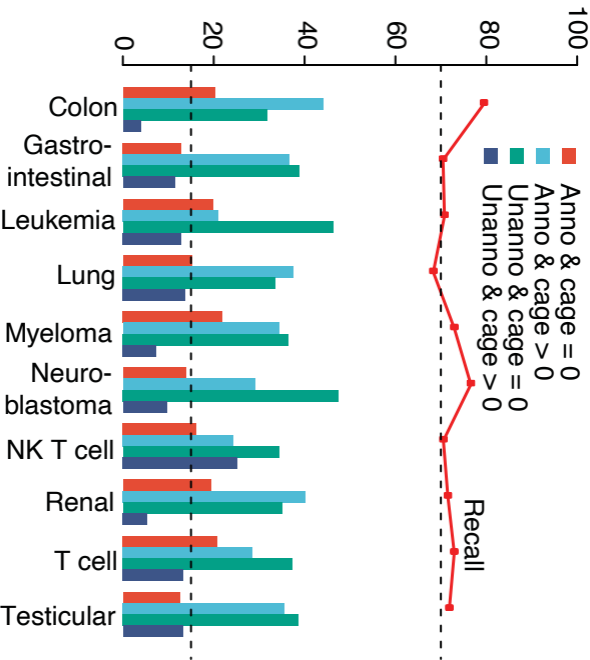

Supplement: Supplementary Figure S3 — DeeReCT-TSS is capable of predicting cell type-specific TSSs across 10 cell types Barplot showing the percentage of four groups of false predicted TSSs. Four groups are the same as Figure S1B. The line above the bar indicates the recall of the prediction in each cell line, while the dashed line indicates 70%. [file mmc4.pdf]
