# Supplementary material for: Annotating TSSs in Multiple Cell Types Based on DNA Sequence and RNA-seq Data via DeeReCT-TSS
Source: Genomics Proteomics Bioinformatics. 2022 Dec 15;20(5):959–73. doi: 10.1016/j.gpb.2022.11.010 (PMC10025762; doi:10.1016/j.gpb.2022.11.010)
Supplement: Supplementary Table S1 — The number of TSSs and the corresponding genes from each cell line used in this study [file mmc5.docx]

| **Metrics** | **No. of active TSSs** | **No. of active genes** | **No. of total TSSs** | **No. of total genes** |
| --- | --- | --- | --- | --- |
| Natural killer T cell leukemia cell line | 24,430 | 10,265 | 47,642 | 14,392 |
| Colon carcinoma cell line | 19,986 | 10,482 | 38,209 | 14,513 |
| Gastrointestinal carcinoma cell line | 26,105 | 11,198 | 53,113 | 16,006 |
| Acute lymphoblastic leukemia cell line | 29,348 | 10,631 | 51,909 | 14,741 |
| Lung carcinoma cell line | 28,325 | 11,482 | 52,944 | 16,365 |
| Myeloma cell line | 21,379 | 10,127 | 41,985 | 14,378 |
| Neuroblastoma cell line | 26,458 | 11,443 | 50,151 | 15,963 |
| Renal carcinoma cell line | 22,493 | 11,046 | 45,023 | 15,653 |
| Adult T cell leukemia cell line | 24,924 | 10,592 | 49,537 | 15,409 |
| Testicular germ cell line | 30,921 | 11,944 | 59,602 | 17,324 |

**Table S1 The number of TSSs and the corresponding genes from each cell line used in this study**
