# Supplementary material for: Annotating TSSs in Multiple Cell Types Based on DNA Sequence and RNA-seq Data via DeeReCT-TSS
Source: Genomics Proteomics Bioinformatics. 2022 Dec 15;20(5):959–73. doi: 10.1016/j.gpb.2022.11.010 (PMC10025762; doi:10.1016/j.gpb.2022.11.010)
Supplement: Supplementary Table S2 — Performance of deep learning-based model on TSS binary classification [file mmc6.docx]

**Table S2 Performance of deep learning-based model on TSS binary classification**

| **Cell line** | **Metrics** | **Integrated model** | **Sequence only model** | **Coverage only model** |
| --- | --- | --- | --- | --- |
| Colon carcinoma cell line | Accuracy | 0.93859 | 0.91493 | 0.77006 |
|  | Recall | 0.90855 | 0.89044 | 0.78362 |
|  | FDR | 0.03337 | 0.06372 | 0.23706 |
|  | F1-score | 0.93669 | 0.9128 | 0.77314 |
| Adult T cell leukemia cell line | Accuracy | 0.90055 | 0.87281 | 0.79748 |
|  | Recall | 0.85616 | 0.82927 | 0.76909 |
|  | FDR | 0.06042 | 0.09163 | 0.18461 |
|  | F1-score | 0.89593 | 0.86702 | 0.79156 |
| Renal carcinoma cell line | Accuracy | 0.91836 | 0.88436 | 0.79715 |
|  | Recall | 0.8518 | 0.82559 | 0.76682 |
|  | FDR | 0.01739 | 0.06444 | 0.18365 |
|  | F1-score | 0.91253 | 0.87713 | 0.79081 |
