# Supplementary material for: Annotating TSSs in Multiple Cell Types Based on DNA Sequence and RNA-seq Data via DeeReCT-TSS
Source: Genomics Proteomics Bioinformatics. 2022 Dec 15;20(5):959–73. doi: 10.1016/j.gpb.2022.11.010 (PMC10025762; doi:10.1016/j.gpb.2022.11.010)
Supplement: Supplementary Table S3 — Performance of DeeReCT-TSS on genome-wide TSSs scanning [file mmc7.docx]

**Table S3 Performance of DeeReCT-TSS on genome-wide TSSs scanning**

| **Cell type** | **No. of used TSSs in the cell type** | **No. of used TSSs detected by integrated model** | **No. of used TSSs detected by sequence only model** | **No. of unused TSSs detected by integrated model** | **No. of unused TSSs detected by sequence only model** | **No. of TSSs not overlapping with FANTOM by integrated model** | **No. of TSSs not overlapping with FANTOM by sequence only model** |
| --- | --- | --- | --- | --- | --- | --- | --- |
| Colon | 17,725 | 14,842 | 14,049 | 4409 | 7708 | 3756 | 30,100 |
| Renal | 19,721 | 15,326 | 13,289 | 5412 | 14,053 | 6018 | 138,383 |
| T cell | 22,171 | 16,900 | 15,477 | 4819 | 12,461 | 6115 | 98,753 |

*Note*: FANTOM, Functional Annotation of The Mammalian Genome.
