# Supplementary material for: Annotating TSSs in Multiple Cell Types Based on DNA Sequence and RNA-seq Data via DeeReCT-TSS
Source: Genomics Proteomics Bioinformatics. 2022 Dec 15;20(5):959–73. doi: 10.1016/j.gpb.2022.11.010 (PMC10025762; doi:10.1016/j.gpb.2022.11.010)
Supplement: Supplementary Table S4 — Performance of TSSs prediction on five T cell datasets with different sequencing depths [file mmc8.docx]

**Table S4 Performance of TSSs prediction on five T cell datasets with different sequencing depth**

| **Depth of RNA-seq** | **No. of ground truth TSSs** | **No. of predicted clusters** | **No. of ground truth TSSs overlapping with predictions** | **No. of predictions overlapping with ground truth TSSs** | **Recall** |
| --- | --- | --- | --- | --- | --- |
| 132 M | 24,924 | 17,690 | 17,525 | 9661 | 0.70 |
| 100 M | 24,924 | 16,847 | 17,361 | 9566 | 0.69 |
| 50 M | 24,924 | 15,096 | 16,919 | 9266 | 0.68 |
| 10 M | 24,924 | 10,356 | 14,118 | 7401 | 0.57 |
| 5 M | 24,924 | 7836 | 11,508 | 5836 | 0.46 |
