# Supplementary material for: Annotating TSSs in Multiple Cell Types Based on DNA Sequence and RNA-seq Data via DeeReCT-TSS
Source: Genomics Proteomics Bioinformatics. 2022 Dec 15;20(5):959–73. doi: 10.1016/j.gpb.2022.11.010 (PMC10025762; doi:10.1016/j.gpb.2022.11.010)
Supplement: Supplementary Table S5 — Performance TSSs prediction on mouse dataset by applying pre-trained DeeReCT-TSS model based on human dataset [file mmc9.docx]

**Table S5 Performance of TSSs prediction on mouse dataset by applying pre-trained DeeReCT-TSS model based on human dataset**

| **No. of ground truth TSSs** | **No. of predicted clusters** | **No. of ground truth TSSs overlapping with predictions** | **No. of predictions overlapping with ground truth TSSs** | **Recall** |
| --- | --- | --- | --- | --- |
| 12,816 | 19,218 | 7034 | 3704 | 0.55 |
